# Up-to-date estimates of long-term cancer survival in England and Wales

**DOI:** 10.1038/sj.bjc.6600976

**Published:** 2003-07-01

**Authors:** L K Smith, P C Lambert, D R Jones

**Affiliations:** 1 Department of Epidemiology and Public Health, University of Leicester, 22-28 Princess Road West, Leicester LE1 6TP, UK

**Keywords:** period survival, cancer relative survival

## Abstract

Cancer survival in England and Wales has improved over the last 30 years. However, cohort survival estimates delay recognition of these improvements. Here we show that period survival estimates, based on survival in a recent time period, suggest a more optimistic pattern for England and Wales than cohort-based measures for most cancers.

Cancer survival in England and Wales has generally improved over the last 30 years. However, conventional survival estimates, based on following-up patient cohorts, delay identification of these improvements. Period analysis is a method of obtaining more up-to-date estimates of survival, calculated using only survival experience in a recent time period (Brenner and Gefeller, 1996). US and European data have shown these estimates to be good predictors of long-term survival (Brenner and Hakulinen, 2001; Brenner, 2002). Here cancer registry data are used to compare 5- and 10-year relative survival estimates for England and Wales using standard cohort methods and period analysis.

## MATERIALS AND METHODS

Data were analysed from the public-use data set of all England and Wales registrations for 1 January 1981 to 31 December 1990 of the 26 most common cancers (Coleman *et al*, 1999) (followed until 31 December 1995). We present 5- and 10-year relative survival estimates for all ages using conventional cohort-based methods and period analyses. Results are presented for males and females combined, apart from results for prostate, testis and gynaecological cancers.

A cohort-based analysis is defined by the time interval in which patients are diagnosed. These patients (and only these) are followed up for 5 or 10 years and survival estimates are calculated. Here cohort estimates for 5 and 10 years are based on patients *diagnosed* between 1986–1990 and 1981–1985, respectively, and followed up until 1995 (see Table 1

**Table 1:** Intervals of diagnosis and follow-up included in estimates of 5- and 10 year relative survival for cohort and period analysis

	**Cohort analysis**		**Period analysis**	
**Survival**	**Diagnosis**	**Follow-up**	**Diagnosis**	**Follow-up**
				
5 years	1986–1990	1986–1995	1986–1990	1990–1995
10 years	1981–1985	1981–1995	1981–1990	1990–1995

). In contrast, period analyses are defined by a recent time interval in which patients' survival experience is observed. It excludes short-term survival of patients diagnosed before the start of the period but includes their long-term survival within the period. Short-term survival of more recently diagnosed patients is included (see Brenner and Gefeller (1996)). Period estimates for 5 and 10 years are based on patients diagnosed between 1986–1990 and 1981–1990, respectively, but only include survival experience from 1990 to 1995 (Table 1).

Survival rates presented here are *relative* survival rates, adjusting for the general population background mortality. We calculated the expected survival using Hakulinen's (1982) method with 95% confidence intervals. A publicly available macro was used to calculate both cohort and period estimates (Brenner *et al*., 2002).

## RESULTS

Table 2

**Table 2:** Five and 10- year **relative survival estimates** (and 95% confidence intervals) for England and Wales, by cancer site using cohort and period survival methods

	**10 years**		**5 years**	
	**Cohort**	**Period**	**Cohort**	**Period**
**Diagnosed**	**1981–1985**	**1981–1990**	**1986–1990**	**1986–1990**
**Followed up**	**1981–1995**	**1990–1995**	**1986–1995**	**1990–1995**
**Cancer site**				
Oral cavity and pharynx	**29.4%**	**30.3%**	**37.6%**	**37.7%**
	28.1%,30.7%	28.7%,31.9%	36.4%,38.8%	35.9%,39.5%
Oesophagus	**6.2%**	**5.3%**	**7.3%**	**5.8%**
	5.7%,6.7%	4.8%,5.8%	6.9%,7.7%	5.3%,6.3%
Stomach	**9.1%**	**9.4%**	**11.1%**	**10.2%**
	8.7%,9.5%	8.9%,9.9%	10.8%,11.4%	9.7%,10.7%
Colon	**34.8%**	**39.2%**	**40.3%**	**42.0%**
	34.3%,35.3%	38.5%,39.9%	39.8%,40.8%	41.3%,42.7%
Rectum	**32.3%**	**34.6%**	**38.7%**	**39.5%**
	31.7%,32.9%	33.8%,35.4%	38.1%,39.3%	38.7%,40.3%
Liver	**3.1%**	**2.7%**	**3.6%**	**3.0%**
	2.3%,3.9%	1.9%,3.5%	2.9%,4.3%	2.2%,3.8%
Gallbladder	**7.5%**	**8.2%**	**10.5%**	**9.2%**
	6.5%,8.5%	6.9%,9.5%	9.5%,11.5%	7.8%,10.6%
Pancreas	**2.7%**	**1.4%**	**3.1%**	**1.6%**
	2.4%,3%	1.2%,1.6%	2.8%,3.4%	1.4%,1.8%
Larynx	**54.8%**	**55.5%**	**62.3%**	**63.0%**
	53.2%,56.4%	53.6%,57.4%	61%,63.6%	61.2%,64.8%
Lung	**5.5%**	**4.4%**	**6.4%**	**5.2%**
	5.3%,5.7%	4.2%,4.6%	6.3%,6.5%	5%,5.4%
Melanoma of the skin	**65.4%**	**72.7%**	**77.3%**	**77.4%**
	64.2%,66.6%	71.5%,73.9%	76.5%,78.1%	76.3%,78.5%
Breast	**50.6%**	**56.4%**	**67.1%**	**68.7%**
	50.2%,51%	56%,56.8%	66.8%,67.4%	68.3%,69.1%
Cervix	**54.2%**	**60.4%**	**63.1%**	**64.6%**
	53.4%,55%	59.3%,61.5%	62.3%,63.9%	63.5%,65.7%
Uterus	**68.6%**	**69.9%**	**71.2%**	**71.7%**
	67.6%,69.6%	68.5%,71.3%	70.3%,72.1%	70.4%,73%
Ovary	**26.1%**	**27.4%**	**30.2%**	**30.4%**
	25.4%,26.8%	26.4%,28.4%	29.5%,30.9%	29.4%,31.4%
Vagina and vulva	**49.0%**	**49.2%**	**52.4%**	**51.7%**
	46.8%,51.2%	46.3%,52.1%	50.6%,54.2%	49%,54.4%
Prostate	**27.9%**	**28.6%**	**42.0%**	**42.3%**
	27.2%,28.6%	27.9%,29.3%	41.4%,42.6%	41.6%,43%
Testis	**87.7%**	**91.0%**	**90.6%**	**91.6%**
	86.5%,88.9%	89.7%,92.3%	89.7%,91.5%	90.3%,92.9%
Bladder	**55.0%**	**56.2%**	**62.1%**	**62.4%**
	54.2%,55.8%	55.3%,57.1%	61.5%,62.7%	61.6%,63.2%
Kidney	**31.4%**	**34.0%**	**38.5%**	**39.7%**
	30.3%,32.5%	32.7%,35.3%	37.6%,39.4%	38.4%,41%
Brain	**9.5%**	**10.5%**	**15.4%**	**14.1%**
	8.9%,10.1%	9.7%,11.3%	14.7%,16.1%	13.1%,15.1%
Thyroid	**66.6%**	**75.3%**	**72.4%**	**76.2%**
	64.4%,68.8%	72.3%,78.3%	70.6%,74.2%	73.4%,79%
Non-Hodgkin's lymphoma	**35.4%**	**38.7%**	**45.5%**	**46.4%**
	34.5%,36.3%	37.6%,39.8%	44.8%,46.2%	45.3%,47.5%
Hodgkin's disease	**63.2%**	**71.3%**	**73.9%**	**76.1%**
	61.7%,64.7%	69.3%,73.3%	72.6%,75.2%	74.2%,78%
Multiple myeloma	**8.0%**	**9.4%**	**20.0%**	**21.0%**
	7.3%,8.7%	8.6%,10.2%	19.1%,20.9%	19.8%,22.2%
All leukaemias	**16.4%**	**19.6%**	**27.8%**	**29.1%**
	15.7%,17.1%	18.6%,20.6%	27%,28.6%	28%,30.2%

Rates derived from England and Wales data for patients diagnosed 1981–1990 (Coleman *et al*, 1999).

shows that period estimates were higher than cohort estimates for 22 (85%) of the 26 cancers for 10-year survival and 18 (69%) of the 26 cancers for 5-year survival. This indicates recent changes in survival that are not detected by standard cohort analyses. At 10 years, period estimates exceeded cohort estimates by over 4% for colon, melanoma of the skin, breast, cervix, thyroid and Hodgkin's disease. Differences at 5 years were less marked but largest differences were seen for thyroid and Hodgkin's disease. Larger differences between cohort and period estimates were seen among cancers with better outcomes (10-year survival >50%). In contrast, where survival was poor (10-year survival <10%), differences between the estimates were smaller and for four of these six cancers, survival estimates were lower for period analysis.

## DISCUSSION

These results provide the most up-to-date estimates of cancer survival in England and Wales. Period survival estimates suggest a more optimistic pattern of cancer survival than cohort-based measures for the majority of cancers despite variations in survival. These differences appear greater for cancers with better outcomes. Our results show similar increases in survival estimates using period analysis as seen in the USA (Brenner, 2002), but with differing site-specific patterns.

Period estimates are similar to cohort estimates where cancer survival patterns have not changed over time. Where survival has changed, period estimates are more up-to-date. Cohort estimates are more influenced by short-term survival of patients diagnosed earlier in a study. If short-term survival changes over time, period analysis allows for these differences.

Since these data represent cancers diagnosed a decade ago, patterns of cancer survival are likely to have changed further. Period analyses of more recent data, when available, are likely to provide the earliest estimates of these developing patterns.
